# Acute Posterior Cranial Fossa Hemorrhage—Is Surgical Decompression Better than Expectant Medical Management?

**DOI:** 10.1007/s12028-015-0217-7

**Published:** 2016-04-12

**Authors:** M. S. Luney, S. W. English, A. Longworth, J. Simpson, S. Gudibande, B. Matta, R. M. Burnstein, T. Veenith

**Affiliations:** 1 School of Clinical Medicine, Addenbrooke’s Hospital, Cambridge, UK; 2 Department of Medicine (Critical Care), The Ottawa Hospital, Ottawa, Canada; 3 Neurosciences Critical Care Unit, Addenbrooke’s Hospital, Cambridge, UK; 4 Critical Care Unit, University Hospital Birmingham NHS Foundation Trust, Edgbaston, UK

**Keywords:** Infratentorial hemorrhage, Cerebellar hemorrhage, Critical care, External ventricular drain, Suboccipital decompressive craniectomy, Intensive care, Tracheostomy

## Abstract

**Background:**

To compare the in-hospital mortality and institutional morbidity from medical therapy (MT), external ventricular drainage (EVD) and suboccipital decompressive craniectomy (SDC) following an acute hemorrhagic posterior cranial fossa stroke (PCFH) in patients admitted to the neurosciences critical care unit (NCCU). Retrospective observational single-center cohort study in a tertiary care center. All consecutive patients (*n* = 104) admitted with PCFH from January 1st 2005–December 31st 2011 were included in the study.

**Methods:**

All patients with a PCFH were identified and confirmed by reviewing computed tomography of the brain reported by a specialist neuroradiologist. Management decisions (MT, EVD, and SDC) were identified from operative notes and electronic patient records.

**Results:**

Following a PCFH, 47.8 % (*n* = 11) patients died after EVD placement without decompression, 45.7 % (*n* = 16) died following MT alone, and 17.4 % (*n* = 8) died following SDC. SDC was associated with lower mortality compared to MT with or without EVD (*χ* ^2^ test *p* = 0.006, *p* = 0.008). Age, ICNARC score, brain stem involvement, and hematoma volume did not differ significantly between the groups. There was a statistically significant increase in hydrocephalus and intraventricular bleeds in patients treated with EVD placement and SDC (*χ* ^2^ test *p* = 0.02). Median admission Glasgow Coma Scale scores for the MT only, MT with EVD, and SDC groups were 8, 6, and 7, respectively (ranges 3–15, 3–11 and 3–13) and did not differ significantly (Friedman test: *p* = 0.89). SDC resulted in a longer NCCU stay (mean of 17.4 days, standard deviation = 15.4, *p* < 0.001) and increased incidence of tracheostomy (50 vs. 17.2 %, *p* = 0.0004) compared to MT with or without EVD.

**Conclusions:**

SDC following PCFH was associated with a reduction in mortality compared to expectant MT with or without EVD insertion. A high-quality multicenter randomized control trial is required to evaluate the superiority of SDC for PCFH.

## Introduction

The posterior cranial fossa (PCF) is the deepest and most confined space in the skull. It has a limited ability to accommodate an expansion of its contents. PCF hemorrhage (PCFH) accounts for between 9 and 15 % of all intracranial hemorrhage in Europe and North America [1–3]. When left to its natural course, it can result in life-threatening complications such as compression of the pons and medulla, resulting in impaired consciousness, respiratory failure, and lower cranial nerve dysfunction. Acute hydrocephalus may also result due to the obstruction of the fourth ventricle leading to herniation of the PCF contents.

The reported mortality from PCFH varies widely and can range from 20 to 75 %, [2, 4–9] depending on the center. The survivors have significant morbidity requiring long-term institutional care. Although management in neurocritical care units have reduced these complications, there is a paucity of evidence to support the current guidelines to manage PCFH [3, 10–13]. Surgical interventions such as suboccipital decompression (SDC) of the PCF have been shown to reduce mortality in well-conducted prospective studies [2]. Bilateral SDC is documented to be safe in patients with malignant cerebellar edema following PCFH, and long-term outcomes following surgery were acceptable [14]. Despite various clinical guidelines, there is institutional variability in the management of PCFH [15].

The aim of our study is to investigate the epidemiology, institutional mortality, and morbidity in patients with PCFH managed in a tertiary center neurosciences critical care unit (NCCU).

## Materials and Methods

### Setting

All patients admitted to the Neurosciences’ Critical Care Unit (NCCU) at Cambridge University Hospitals NHS Foundation Trust with a PCFH from January 1st 2005 to December 31st 2011 were included in this study. This study was reviewed by Cambridge East Research Ethics Committee, and a formal review by ethics committee was waived.

### Patient Identification

Addenbrooke’s Hospital NCCU is a 21-bed intensive care unit specializing in the care of patients with neurological disorders. Patients are managed by dedicated neurocritical care physicians, nurses, and allied health care professionals. All NCCU admissions are prospectively logged in the intensive care national audit and research centre (ICNARC) database which includes primary diagnosis, demographic, and clinical data. This high-quality national database is maintained by a multidisciplinary team which includes research nurses and database administrators. The quality of the accrued data is routinely checked and verified by independent audits to compare our institutional performance with other intensive care units in the United Kingdom.

All patients admitted between January 1st 2005–December 31st 2012 with a primary neurosurgical or neurology admitting team were considered for inclusion. Patients admitted with a non-PCF stroke were excluded; in remaining patients, the brain computed tomogram (CT) and subsequent report by a neuroradiologist were reviewed. All patients with the evidence of a PCFH on CT scan were included in this study. In patients where the diagnosis was unclear, neuroimaging was reviewed by a second (SWE) and a third reviewer (TV) to reach a consensus. Patients with a primary subarachnoid hemorrhage, trauma, or malignancy were excluded from this study.

Descriptive data such as patient demographics and baseline Glasgow Coma Scale (GCS) score were retrieved from the ICNARC database. Electronic discharge, operative notes, and death summaries when available were also reviewed. Primary and secondary outcome data were obtained from the information collected on the ICNARC database.

### Outcomes

Our objective was to compare medical management (MT) with or without external ventricular drain (EVD) to SDC. Our primary outcome measures of interest were NCCU and in-hospital mortality. Morbidity was assessed using NCCU and hospital length of stay as well as the incidence of tracheostomy.

### Statistical Analysis

Statistical analyses were conducted using Statview (Version 5, 1998, SAS Institute Inc., Cary, North Carolina, USA). Paired non-parametric data were compared using the Mann–Whitney *U* test, and non-parametric data with three or more datasets were compared using the Freidman test. Appropriate Bonferroni corrections were applied, and a *p* value of <0.05 was considered statistically significant.

## Results

### Patient Characteristics

Between January 1st 2005 and December 31st 2011, 5915 patients were admitted to the NCCU. Once our exclusion criteria were applied, we identified 104 patients with PCFH. Emergency admissions to Addenbrooke’s Hospital accounted for 48.1 % of PCFH patients (*n* = 50). The remaining 51.9 % (*n* = 54) were secondary referrals from other hospitals.

The demographic and baseline clinical characteristics of patients with PCFH are summarized in Table 1. Admission GCS of MT patients with or without EVD did not differ significantly (*p* = 0.11). GCS did not differ in patients managed surgically compared to MT with (*p* = 0.40) or without EVD (*p* = 0.25). The GCS could not be ascertained for 28 patients (26.9 %) due to sedation upon NCCU admission. Age, ICNARC score, and brain stem involvement did not differ significantly between groups. There was no significant progression in initial CT hematoma volume compared to 24 h post admission. At 24 h, the mean volume in milliliters for MT was 7.1, 7 mls for MT with EVD, and SDC 12 mls (*p* = 0.45). However, there was a statistically significant presence of hydrocephalus and intraventricular hemorrhage in patients treated with EVD and SDC (*p* < 0.05).

**Table 1:** Patient characteristics

	Overall	MT	EVD	SDC	*p* value
Number of patients (%)	104 (100)	35 (34)	23 (22)	46 (44)	–
Male (%)	64 (62)	25 (71)	14 (61)	25 (54)	–
Age in years (±SD)	57.7 ± 15.9	56.1 ± 17.4	57.6 ± 17.7	58.8 ± 13.9	0.88
GCS score (range)	7 (3-15)	8 (3-15)	6 (3-11)	7 (3-13)	0.89
ICNARC score (±SD)	18.7 ± 6.8	19.2 ± 8.1	18.7 ± 8.0	18.4 ± 5.1	0.37
Volume of bleed (mls) (±SD)	8.2 ± 8.8	7.1 ± 8.2	7.0 ± 7.6	12 ± 8	0.45
Presence of hydrocephalus (%)	70.0	48.5	75	90.3	0.02*
Presence of IVH (%)	57.0	36.6	85.7	70.5	0.001*
Brain stem involvement (%)	16.1	15.6	13.3	19	0.13

The demographic and clinical characteristics of patients with spontaneous posterior cranial fossa hemorrhage split by management strategy. GCS is defined as the lowest Glasgow coma scale score within first 24 h of admission

*MT* medical therapy without external ventricular drainage; *EVD* medical therapy with EVD; *SDC* surgical decompressive craniotomy; *SD* standard deviation; *IVH* intraventricular hemorrhage

* Statistically significant

### Primary Outcome

Of the 104 patients studied, 69 (66.3 %) survived their admission from the NCCU to discharge home or to a rehabilitation unit. Thirty one patients died in NCCU and a further four following discharge from NCCU. Survival to discharge or death in the hospital is summarized in Table 2. Mortality was the lowest in the surgically treated patients and those receiving medical therapy with EVD insertion had higher mortality compared to surgical decompression (47.8 vs. 17.4 %, *p* = 0.008, OR = 4.4, RR = 2.8) and medical therapy without EVD (45.7 vs. 17.4 %, *p* = 0.006, OR = 4.0, RR = 2.6). In surgically managed patients, the mean time to surgical decompression was less than 24 h.

**Table 2:** Hospital survival

	Overall	MT	EVD	SDC
Alive (%)	69 (66.3)	19 (54.3)	12 (52.2)	38 (82.6)
Dead (%)	35 (33.7)	16 (45.7)	11 (47.8)	8 (17.4)

The proportion of patients surviving to discharge following PCFH with admission to NCCU stratified by management strategy

*MT* medical therapy only; *EVD* external ventricular drain, *SDC* suboccipital decompressive craniectomy; *SD* standard deviation

### Patient Characteristics Based on Mortality

Admission GCS and ICNARC scores differed significantly between PCFH survivors and non- survivors (see Table 3). In the subgroup of patients with an admission GCS less than 8, a higher proportion of surgically managed patients survived (73.7 %) compared to MT patients (26.9 %) This was inclusive of patients treated with an EVD. However, there was no significant difference in mean GCS (*p* = 0.40), ICNARC score (*p* = 0.44), and age (*p* = 0.44). Baseline GCS was not significantly different in MT patients without EVD compared to patients with an EVD (*p* = 0.91).

**Table 3:** Patient characteristics based on mortality

	Overall	Alive	Dead	*p* value
Number of patients (%)	104 (100)	69 (66.3)	35 (33.7)	–
Male (%)	64 (62)	38 (55)	26 (74)	–
Age in years (±SD)	57.7 ± 15.9	56.1 ± 15.9	60.8 ± 15.7	0.15
GCS score (range)	7 (3-15)	8 (3-15)	4 (3-9)	<0.0001
ICNARC score (±SD)	18.7 ± 6.8	16.7 ± 5.9	22.8 ± 6.6	<0.0001

Demographics and clinical characteristics of patients with acute non-traumatic PCF hemorrhage based on primary outcome

### Secondary Outcomes

**Fig. 1:**
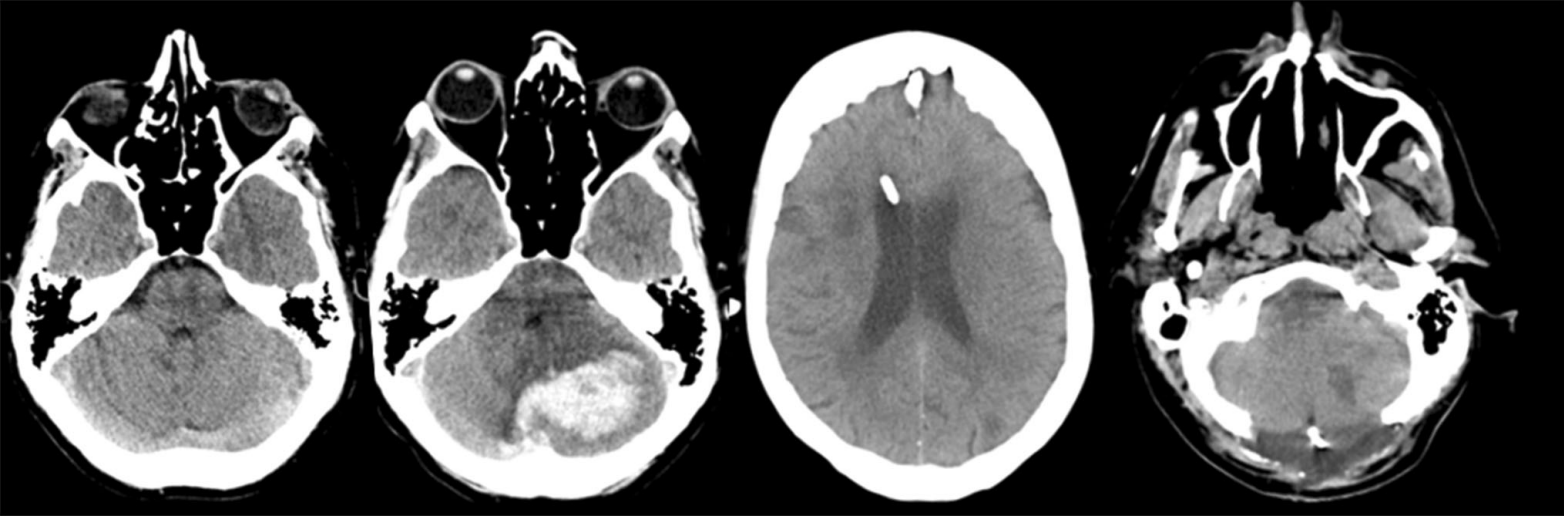
Computed tomography showing the head of a patient admitted with a posterior cranial fossa bleed. This patient was initially treated with external ventricular drain followed by posterior cranial fossa decompression

An EVD was inserted in 93.5 % (*n* = 43) of patients undergoing decompression compared to 39.7 % (*n* = 23) of patients managed non-surgically. Surgically managed patients had a longer NCCU stay compared to those with MT, including patients with an EVD (17.4 ± 15.4 versus 7.2 ± 8.8 (mean ± SD) respectively, *p* < 0.001, 95 % CI −14.9, −5.4). Hospital length of stay was longer in surgically managed patients (42.2 ± 53.5 versus. 21.7 ± 28.0 (mean ± SD), *p* = 0.013, 95 % CI −36.6, −4.3) (Table 4). MT managed patients surviving PCFH experienced similar lengths of stay in hospital, regardless of EVD insertion (*p* = 0.32). Surgically managed patients had a longer hospital stay compared to those medically managed with or without an EVD. However, this did not reach statistical significance (*p* = 0.088), despite inclusion of those with an EVD (*p* = 0.794). Surgical decompression (Fig. 1) resulted in a higher incidence of tracheostomy compared to MT with or without an EVD (50 vs. 17.2 %, *p* = 0.0004).

**Table 4:** Secondary outcomes from PCF hemorrhage

	Overall	MT	EVD	SDC
Mean length of stay (d) (±SD)				
NCCU, all	11.7 ± 13.1	6.0 ± 7.7	9.1 ± 10.3	17.4 ± 15.4
NCCU, survivors	15.2 ± 14.0	8.0 ± 7.9	12.3 ± 12.2	20.3 ± 15.4
NCCU, non-survivors	3.7 ± 5.1	2.6 ± 6.2	5.0 ± 5.4	3.7 ± 2.6
Hospital, all	30.8 ± 42.2	21.6 ± 30.3	22.0 ± 24.7	42.2 ± 53.5
Hospital, survivors	41.3 ± 46.2	26.4 ± 30.1	37.0 ± 26.1	50.1 ± 55.5
Hospital, non-survivors	10.1 ± 21.3	15.9 ± 30.6	5.5 ± 4.9	4.8 ± 3.7
Tracheostomy: number of patients, (%)	33 (31.7)	4 (11.4)	6 (26.1)	23 (50)

*MT* medical therapy only; *EVD* external ventricular drain; *SDC* suboccipital decompressive craniectomy; *d* days; *SD* standard deviation; *NCCU* neurosciences critical care unit

## Discussion

47.1 % of patients admitted to a tertiary center NCCU following PCFH underwent SDC.

SDC was associated with an increased survival in patients compared to MT with or without EVD insertion. Surgery was offered more frequently for patients with hydrocephalus and intraventricular hemorrhage. Baseline GCS, ICNARC scores, and brainstem involvement were similar in all treatment groups. In our study, the use of EVD without surgical decompression compared to medical management alone was associated with an increase in mortality.

SDC is a treatment option for the management of deteriorating patients with acute PCFH and rising PCF pressure [11]. Previous small studies have shown an association of SDC for PCFH and reduced mortality and morbidity, despite low initial GCS [12, 13]. Although recommended in clinical guidelines, evidence is sparse to recommend SDC routinely [2, 16]. Management strategies proposed by current guidelines are mixed [3, 11, 17, 18]. The guidelines for the management of spontaneous intracerebral hemorrhage produced by the American Heart Association Stroke Council [11] recommends surgical removal of hematoma for patients with PCFH and neurologic deterioration, brainstem compression, or hydrocephalus. This guidance has been developed largely from observational and retrospective studies, some of which supports EVD as a viable independent treatment option [19]. These interventions are dependent on institutional guidelines focusing on the degree of 4^th^ ventricle occlusion [20] or hematoma dimensions [2]. A strategy focussed solely on EVD may be ineffective compared to SDC in the management of PCF hemorrhage.

Our data support the use of SDC rather than expectant medical management with or without EVD. The American Stroke Association recommendations favoring SDC were made in 2015 [11] and the management choices may have changed following these recommendations. In this study, a low GCS and ICNARC (a measure of peri-admission morbidity) score were associated with increased mortality. Previous studies are in agreement with our institutional data regarding the importance of GCS as a prognostic marker after PCFH [13, 21]. Our study has demonstrated a trend toward increased survival following SDC, even in patients presenting with low GCS.

Infratentorial lesions may affect lower cranial nerve function leading to tracheostomy. This may be used to demonstrate institutional morbidity and the severity of stroke [22, 23]. Despite improved mortality, the incidence of tracheostomy in surgically managed patients was higher than medically managed patients (50.0 vs. 17.1 %, Table 3). This may be explained by the added edema, injury, and the time required for surgical recovery following decompression of the posterior cranial fossa.

In this study, patients who died had a shorter duration of ICU and hospital stay compared to survivors. This may be related to the withdrawal of care as a response to severe brain injury. This trend is also seen in other patient cohorts, for example, in traumatic brain injury [20]. Longer ICU stay in surviving surgical patients was likely due to surgical recovery time and prolonged weaning from mechanical ventilation [24]. A recent study suggests that these patients may benefit from early tracheostomy to facilitate weaning from mechanical ventilation [25].

One of the strengths of our study is the completeness of data over a period of 7 years and the use of a comprehensive and validated database. Previously described literature has been based on smaller cohorts or a less rigorous case definition [2, 6, 8, 12, 13, 26–28], making this one of the largest studies of its kind. Limitations of our study include the retrospective and non-randomized nature of data used. There is potential for selection bias as our unit is a specialist center within a tertiary care hospital and as such attracts the “unwell” patients requiring intensive care treatment. Futility and patient outcome may have biased the transfer of patients to our tertiary referral center thus skewing results. However, in our study, 51.2 % of patients with PCFH were referrals from other hospitals. In our region, Glasgow coma score was used as a tool to trigger referral in patients with PCFH. In this study, median GCS was 5.5 for patients presenting directly to emergency department and those referred for tertiary management.

There are many other factors not included in our study that could play a role in the management decision to perform or withhold surgical decompression. They include trends in GCS score, cerebral perfusion, pupillary responses, the degree of 4^th^ ventricle collapse, and the presence of cerebral edema. These highlight the need for a carefully planned study incorporating the above factors, regarding the management of PCFH.

In the absence of a volumetric magnetic resonance imaging for our patients, a CT brain was used to measure the volume of bleed. This approach could introduce errors due to partial volume effects and beam-hardening artifacts. The results of our study cannot be generalized to other posterior fossa pathologies including traumatic and spontaneous subarachnoid hemorrhage [20].

Our study only addresses ICU and in-hospital mortality and morbidity and not long-term functional and neurocognitive outcomes. Although our findings suggest that an aggressive surgical approach may be associated with a reduced mortality after PCFH, it remains unclear which patients are best suited to this approach and whether functional morbidity and long-term mortality outcomes are affected as suggested in other centers [8, 24]. Our results clearly demonstrate the requirement for a high-quality multicenter randomized trial (RCT) in patients with PCFH.

## Conclusions

Suboccipital decompression was only undertaken in 47 % of patients with an acute hemorrhage in the posterior cranial fossa following admission to a neurocritical care unit. SDC was associated with a favorable survival compared to medical management alone (with or without EVD insertion) in our tertiary referral hospital. Definitive conclusions cannot be drawn from these data and a high-quality randomized control trial should be performed in this cohort of patients.
